# Chemical Desiccation in the Preharvest of Cowpea: A Study of How the Time of Application Interferes in the Enzymatic and Physiological Aspects of Seedlings from Desiccated Plants

**DOI:** 10.1021/acsomega.4c04489

**Published:** 2024-08-03

**Authors:** Ester dos Santos Coêlho, João Everthon da Silva Ribeiro, Pablo Henrique de Almeida Oliveira, Welder de Araújo Rangel Lopes, Anna Kézia Soares de Oliveira, Matheus de Freitas Souza, Hamurábi Anizio Lins, Clarisse Pereira Benedito, Lindomar Maria da Silveira, Aurélio Paes Barros Júnior, Daniel Valadão Silva

**Affiliations:** † Department of Agronomic and Forestry Sciences, Universidade Federal Rural do Semi-Árido, Mossoro, Rio Grande do Norte 59625-900, Brazil; ‡ Department of Agronomic, Universidade de Rio Verde, Rio Verde, Goias 75901-970, Brazil

## Abstract

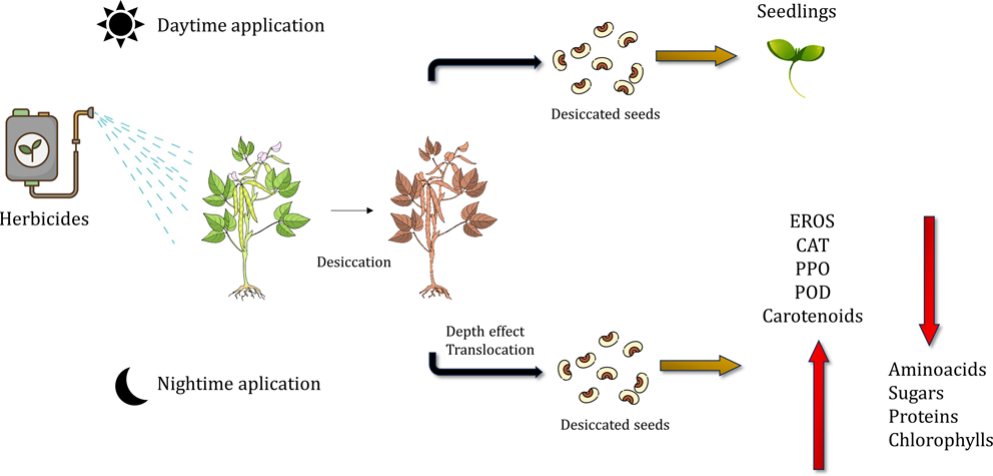

Chemical desiccation in the preharvest of grains and seeds is commonly used in production fields. Using herbicides for this purpose is a viable alternative to reduce beans’ exposure to adverse crop conditions. Our objectives were to evaluate (1) the efficacy of herbicides for accelerated defoliation of cowpea, (2) the impact of herbicide application on antioxidant enzyme activity and protein and amino acid contents in seeds, and (3) the effects of different herbicide application schedules on the physiological aspects of seeds. In the first experiment, in addition to the control treatment (without herbicides), seven herbicides and two mixtures were applied at night: diquat, flumioxazin, diquat + flumioxazin, glufosinate ammonium, saflufenacil, carfentrazone, diquat + carfentrazone, atrazine, and glyphosate. Diquat and its mixtures showed greater efficacy in anticipating the harvest. Flumioxazin and diquat alone reduced amino acid content by 61.72 and 51.44%, respectively. The same trend was observed for total soluble proteins. The activity of antioxidant enzymes (CAT, POD, PPO) increased, indicating oxidative stress caused by diquat and flumioxazin. In the second experiment, we tested three application times (6 a.m., 12 p.m., 6 p.m.) with diquat, diquat + flumioxazin, and diquat + carfentrazone. The lowest damage to chlorophyll a was at 6 a.m.; other times reduced photosynthetic pigments and increased carotenoid content. Total soluble sugars decreased by 27.74% with nocturnal application of diquat + flumioxazin. Our data indicate that herbicide use for desiccation affects seed quality. These findings highlight the need for selecting appropriate herbicides and application times. Future research should explore long-term impacts on crop yield and quality.

## 1 Introduction

Anticipation of harvest is a commonly used practice in seed production fields and succession cropping systems.^1^ In the first situation, the anticipation of the harvest allows for a higher physiological quality of the seeds since it reduces their deterioration due to conditions of biotic and abiotic stresses, such as high rainfall, excessive heat, frost, pest attack and diseases.^2^ In the second scenario, the anticipation increases the planting window for succession crops, enabling, for example, the cultivation of off-season corn in numerous regions of the Brazilian cerrado.^3^

Anticipating the harvest is often unfeasible due to that, many leaves and branches not yet senescent on the plant, restricting mechanical harvesting due to the frequent filling of the harvesting platform.^4^ Thus, applying herbicides moments before harvest is the main strategy to enable the mechanical harvesting of the areas. Once absorbed, the herbicide triggers the leaf abscission process and accelerates the defoliation and senescence of the green branches of the plant.^5^ For seed producers, herbicide application allows mechanized harvesting of seeds when they reach their maximum physiological maturity, allowing seed lots with greater vigor and germination. The rapid security of the branches and leaves, in addition to allowing mechanical harvesting, accelerates the loss of moisture from the seed, a fundamental step in the seed production process.^1^

Preharvest desiccation is often necessary in soybean and bean production fields because some cultivars show more significant variation in seed maturity and the beginning of branch and leaf sequence.^6^ In addition to these crops, cowpea [*Vigna unguiculata* (L.) Walp]^7^ exhibits many nonsenescent branches and leaves after physiological seed maturation. In addition, this crop may present variations in its cycle length, such as prolonging the vegetative stage in some environmental conditions.^8^ Consequently, producing seeds with high vigor and germination can be compromised due to their exposure to stress events.^9^ Thus, applying herbicides for preharvest desiccation becomes crucial for the mechanical harvesting of cowpea seed production fields.^10^

The primary herbicide used for preharvest desiccation on Brazilian agricultural properties was paraquat. Moreover, it acts by inhibiting photosystem I in the thylakoid membrane.^11^ Paraquat has high agronomic efficiency for the desiccation of plants such as soybeans, beans and cowpeas.^12^ However, the use of this herbicide, regardless of the application modality, has been prohibited by health agencies since 2019.^13^ Since then, searching for new efficient alternatives for preharvest desiccation has challenged research institutions. Although other herbicides are registered for preharvest desiccation, their efficiency does not reach levels similar to those of the extinct paraquat. Some alternatives showed greater efficiency for preharvest desiccation, such as saflufenacil for common bean,^14^ glyphosate for wheat^15^ and diquat for soybeans.^16^ However, there are no registered herbicides for preharvest desiccation of cowpea,^17^ and no studies evaluating the physiological quality and biochemical parameters of seeds from plants desiccated with possible herbicides for use in this application.

The evaluation of agronomic efficacy and safety for the use of herbicides for preharvest desiccation in seed production fields should follow some essential criteria, such as applied dose, environmental conditions during application, mechanism of action and translocation of the herbicide, phenological stage of the crop and possible herbicide residues in the seeds that may reduce the vigor and germination of the lots.^18^ In addition to these factors, the time of application can also have a positive or negative influence on the action of herbicides.^19^

The nighttime application provides favorable conditions, such as a decrease in temperature and an increase in relative humidity, in addition to favoring the logistical conditions of the property.^20^ In addition, it reduces the photodegradation of the herbicide molecule.^21^ Cieslik et al.^22^ found that in environments with lower light intensity, under certain specific conditions, there are improvements in herbicide performance. However, the environmental variables behave differently according to the time and day the herbicide is applied. It is complex to decide and understand the best application time and seek the products’ most excellent effectiveness.^23^ Nighttime application increases the translocation of some herbicides and further enhances their action as desiccants.^24^

Among the herbicides with the most significant potential for preharvest desiccation, those with limited translocation, popularly called contact herbicides, have a more significant advantage due to their rapid defoliant action than the others.^25^ In addition, limited translocation reduces the herbicide’s potential to migrate to the seed through symplastic transport pathways. For example, when applied to leaves, systemic herbicides can translocate and accumulate in the seed.^25,26^ This scenario can be unfavorable due to possible damage to the seedling generated from these contaminated seeds, impairing the initial establishment of the crop or even reducing the harvested productivity.

The possible stress caused by herbicide residues infers a reduction in physiological and biochemical properties of seeds, which can generate the production of reactive oxygen species (ROS), alteration in the production of amino acids resulting in protein damage, decrease in photosynthetic pigments, and lipid peroxidation.^27^ Under these conditions, plants contain both enzymatic and nonenzymatic defense components, which involve increased activity of enzymes such as catalase (CAT), peroxidase (POD), and polyphenoloxidase (PPO),^28−30^ the accumulation of osmoregulatory, and the increase of carotenoids.^31,32^

Known studies have investigated seed yield, color, physiological quality, legume germination variables, and application of desiccants.^1,33^ Chamma et al.^34^ found that the application of desiccants for the forced maturation of soybeans influenced the acquisition of vigor and longevity of the seeds. For chickpeas, Almeida et al.^35^ observed that using glufosinate (400 g i.a/ha) anticipated harvest by 17 days and increased germination and vigor in chickpea seeds. Although cowpea is widely cultivated in the Brazilian agricultural scenario, the use of herbicides in the preharvest is widespread, and there are still no studies that portray physiological and biochemical parameters that confer quality to seeds obtained from plants desiccated with herbicides.

Thus, studies aiming to select potential herbicides for preharvest application in cowpea should consider both their efficacy in defoliation and potential oxidative damage to seeds. Therefore, we hypothesize that the choice of herbicide and the timing of application can induce physiological and biochemical changes in seeds of desiccated plants. Specifically, we anticipate that certain herbicides and application timings may increase oxidative stress and decrease seed quality through alterations in antioxidant enzyme activity, as well as reductions in protein and amino acid levels. Conversely, we expect that some herbicide treatments could achieve effective defoliation without significantly compromising seed quality. Our research objectives were (1) to evaluate the efficacy of herbicides for accelerating defoliation of cowpea at physiological maturity, (2) to assess the effects of herbicide application in the preharvest stage on antioxidant enzyme activity, protein content, and amino acid levels in cowpea seeds, and (3) to examine the effects of different herbicide application schedules in the preharvest stage on the physiological characteristics of cowpea seeds.

## 2 Results and Discussion

### 2.1 Experiment I

Only the herbicides carfentrazone and flumioxazin did not anticipate the cowpea harvest. The herbicides diquat, diquat + flumioxazin and diquat + carfentrazone increased the cowpea harvest in 11 days compared to the control (Table 1). The herbicides saflufenacil and glyphosate anticipated harvest by 8 days, while glufosinate and atrazine shortened the time to harvest by only 5 days (Table 1).

**Table 1:** Moisture Content of Freshly Harvested Seeds and Time of Harvest Anticipation under Herbicide Application on Cowpea (BRS-Tumucumaque) Plants

herbicides	moisture degree (%)	harvest anticipation (Days)
diquat	10.7	11
flumioxazin	10.6	0
diquat + flumioxazin	10.8	11
glufosinate	10.6	5
saflufenacil	11.6	8
carfentrazone	10.6	0
diquat + carfentrazone	10.8	11
atrazine	10.6	5
glyphosate	11.6	8
witness	10.5	0

Although the mixtures diquat + flumioxazin and diquat + carfentrazone demonstrate efficiency for anticipating the harvest of cowpea seeds, the isolated application of diquat is sufficient to anticipate the harvest. The presence of flumioxazin or carfentrazone together with diquat did not increase the days of anticipation. On the contrary, applying flumioxazin and carfentrazone did not accelerate the defoliation of cowpea plants, causing only mild symptoms on the leaves (data not shown). Assis et al.^7^ observed that only the application of isolate from another herbicide belonging to the group of photosystem I inhibitors, paraquat, was practical in anticipating the harvest of cowpea by 13 days. The higher efficacy of herbicides that inhibit photosystem I to anticipate harvest may be related to the rapid action of these herbicides. After the leaf uptake process, both diquat and paraquat capture all cellular free electrons generated by the metabolic pathways of respiration and photosynthesis, rapidly triggering the formation of free radicals responsible for membrane peroxidation.^36^ Consequently, symptoms of chlorosis, followed by necrosis, are observed hours after the application of these herbicides to sensitive plants.^37^

The herbicides saflufenacil and glyphosate also anticipated cowpea harvest, but 3 days less compared to treatments with diquat. Glyphosate is a systemic herbicide with a slower action on sensitive plants, justifying the longer time for desiccation of cowpea branches and leaves compared to diquat. However, saflufenacil, like diquat, is a contact herbicide, but the action on defoliation in cowpea was slower. Saflufenacil works by inhibiting the enzyme PROTOX by promoting lipid peroxidation. However, the time to generate the first reactive oxygen radicals occurs only after a series of chain reactions and in the obligatory presence of light.^38^ This process slows down the action of saflufenacil for defoliation relative to diquat. On the other hand, by capturing free electrons generated by both cellular respiration and photosynthesis, diquat already induces the formation of reactive oxygen radicals, initiating the peroxidation process soon after their absorption.

Treatments with the use of diquat were more efficient in anticipating the harvest, which may have resulted from the mode of action of this herbicide, which promotes rapid onset of symptoms and, consequently, the death of the plant occurs in a shorter interval of up to 2 days after its application.^39^ Diquat belongs to the chemical group of bipyridyls and acts directly on the photochemical phase of photosynthesis; it is considered a potent reducing agent that promotes the capture of electrons from PSI.^40^ The free radicals formed by the action of diquat are readily oxidized in the presence of molecular oxygen. Consequently, these are not responsible for the symptoms of toxicity observed.^41^ During this reaction, superoxide radicals undergo dismutation and form hydrogen peroxide (H_2_O_2_) molecules responsible for lipid peroxidation, followed by cell death.^41^

There were no normal seedlings in treatment 7 (diquat + carfentrazone), which made biochemical and enzymatic analyses impossible since healthy green leaves are needed.

**Figure 1:**
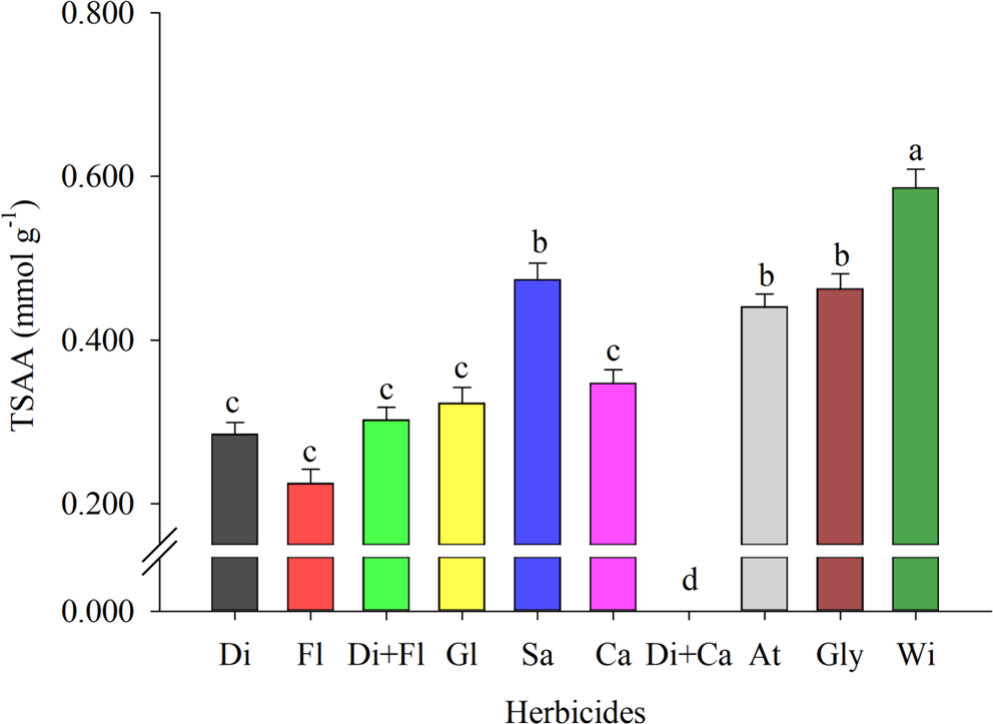
Total soluble amino acids (TSAA) in cowpea (BRS Tumucumaque) seedlings desiccated with herbicides during preharvest. Di: Diquat; Fl: Flumioxazin; Di + Fl: diquat + flumioxazin; Gl: Glufosinate; Sa: Saflufenacil; Ca: Carfentrazone; Di + Ca: diquat + carfentrazone; At: Atrazine; Gly: Glyphosate; Wi: witness. * Means followed by the same letter do not differ from each other using the Scott-Knott test at 5% probability.

Applying desiccant herbicides reduced the total soluble amino acid (TSAA) content (Figure 1). The lowest TSAA values were observed with the application of flumioxazin (T2) and diquat (T1), with a reduction of 61.72 and 51.44%, respectively (Figure 1). In addition, it was also possible to observe a reduction of 48.48% in the treatment with the combination of these two herbicides, diquat + flumioxazin (T3) (Figure 1).

The decrease in TSAA indicates that cowpea plants subjected to applying these herbicides suffered stress, in which the plant’s responses to this condition directly involve amino acid metabolism.^42^ The reduction in TSAA observed for the application of diquat and flumioxazin may have been caused by the use of amino acids as an energy substrate during cellular respiration at the beginning of the germination process and initial growth after the emergence due to the lower availability of sugar in the seed, caused by the photosynthetic blockade caused by the herbicides applied. Carbohydrates are the substrate preferred by the cowpea embryo for energy generation. However, the lower availability of carbohydrates and lower photosynthetic rate caused by the presence of the herbicides flumioxazin, diquat and diquat + flumioxazin in the seed may have stimulated pathways for conversion of amino acids into energy for seedling growth.^43^ Studies show a variation in amino acid content under various conditions of oxidative stress; for example, proline accumulation may occur as a strategy for mitigation and adaptation.^44^ Hildebrandt et al.^45^ state that under conditions of abiotic stresses, amino acids such as proline, glutamine, asparagine, and arginine are synthesized in more significant quantities, being a mitigation strategy. On the other hand, Chen and Hoehenwarter^46^ found in their study that the oxidative stress caused by applying H_2_O_2_ reduced glycine levels, an amino acid abundant in plants. The results found in the present study indicate that under unfavorable conditions of residues that promote toxicity, what can occur is a redistribution of metabolic fluxes as a way to mitigate the effects of ROS and maintain the synthesis of vital compounds.^47^

**Figure 2:**
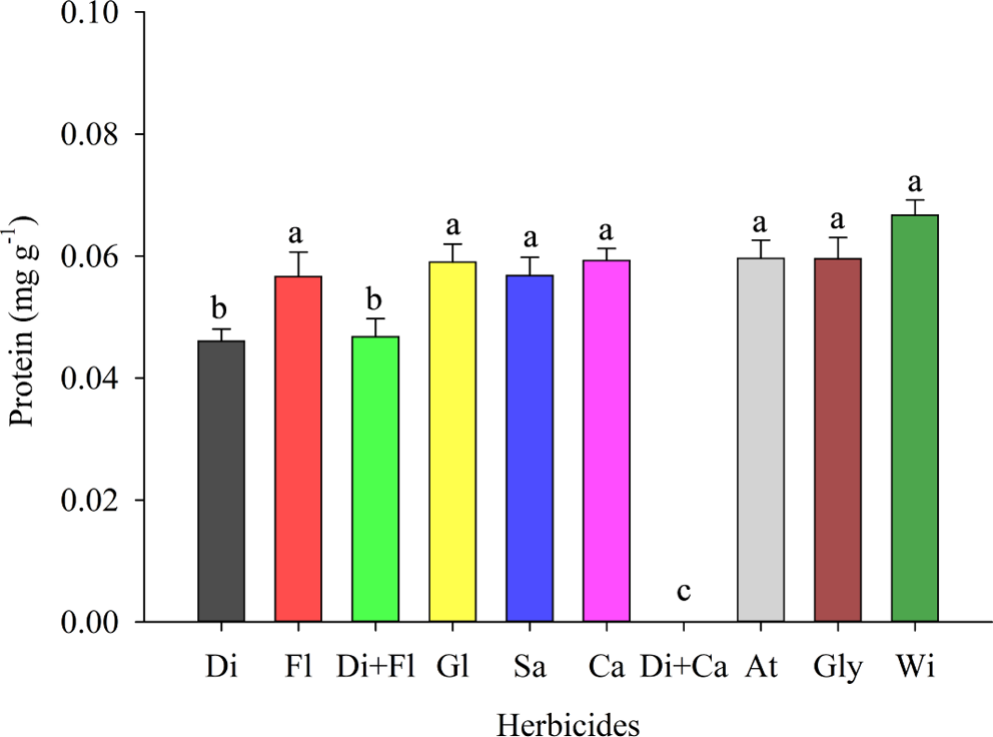
Total soluble protein in cowpea (BRS Tumucumaque) seedlings desiccated with herbicides in preharvest. Di: Diquat; Fl: Flumioxazin; Di + Fl: diquat + flumioxazin; Gl: Glufosinate; Sa: Saflufenacil; Ca: Carfentrazone; Di + Ca: diquat + carfentrazone; At: Atrazine; Gly: Glyphosate; Wi: witness. * Means followed by the same letter do not differ from each other using the Scott-Knott test at 5% probability.

The total soluble protein (TSP) followed the same trend as the total soluble amino acids (TSAA), where it was possible to observe that there is a reduction in the TSP under the application of diquat (T1), flumioxazin (T2) and diquat + flumioxazin (T3) (Figure 2). The decrease was 30.97, 15.08, and 29.89%, respectively.

The negative impact of herbicides on TSP is explained by the decrease in TSAA in similar treatments (T1, T2 and T3), demonstrating that the reduction in amino acids causes a severe effect on protein synthesis. This reduction in protein synthesis can be explained by the change in cytochrome oxidase activity caused by herbicides, which limits the airways and causes the production of succinate.^48^ Proteins play essential roles in plant metabolism, and ROS causes changes in the functioning and content of lipids, nucleic acids, and proteins.^49^ Sachdev et al.^50^ state in their study that pesticide-induced stress is known to cause oxidative damage, contributing to the formation of EROS. These reactive molecules are responsible for causing this reduction in protein synthesis.^51^ Diquat is a herbicide commonly used in desiccating grain crops and legumes. This herbicide acts on reduction–oxidation reactions, producing free radicals that interfere with the vital processes of plants, including protein synthesis.^52^

**Figure 3:**
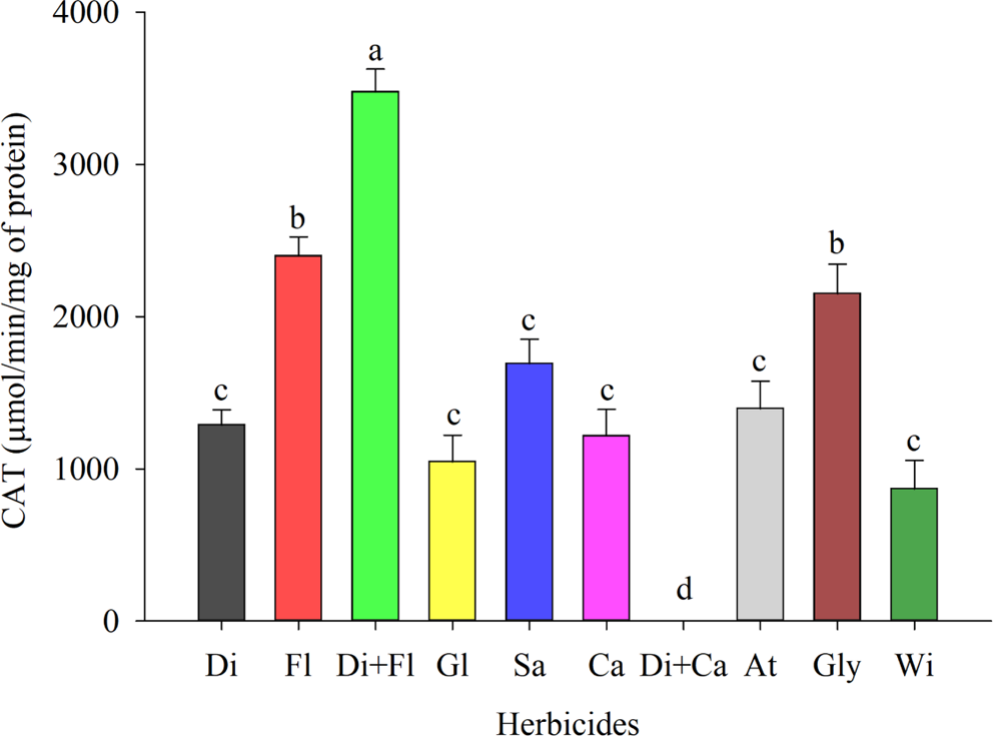
Catalase (CAT) in cowpea (BRS Tumucumaque) seedlings desiccated with herbicides in preharvest. Di: Diquat; Fl: Flumioxazin; Di + Fl: diquat + flumioxazin; Gl: Glufosinate; Sa: Saflufenacil; Ca: Carfentrazone; Di + Ca: diquat + carfentrazone; At: Atrazine; Gly: Glyphosate; Wi: witness. * Means followed by the same letter do not differ from each other using the Scott-Knott test at 5% probability.

The application of desiccant herbicides increased the activity of the catalase enzyme (Figure 3). The catalase activity (CAT) was higher in the treatment containing diquat + flumioxazin (T3), with an increase of 75.04% compared to the control (Figure 3). Flumioxazin (T2) provided a 63.83% increased CAT activity (Figure 3). The highest levels of enzymatic activity were detected in the herbicides mentioned; in the others, it tended to decrease, and the lowest value was found in the control treatment (Figure 3). It was observed that herbicides with a more severe effect on TSAA and TSP also caused higher CAT activity.

The results confirmed the relationship between CAT and the attempt to neutralize plant herbicides as a defense strategy (Peterson et al., 2016). Therefore, the application of these herbicides promotes an increase in CAT activity. The increase observed in the present study can be compared with the results observed by Jiang et al.,^53^ Zhang et al.,^54^ Boulahia et al.^55^ and Pan et al.,^56^ who found that the exposure of crops such as soybeans, rice, beans and wheat to herbicides promotes more excellent production of reactive oxygen species and, Consequently, the synthesis of antioxidant enzymes such as catalase. This increase denotes the presence of ROS and possible oxidative damage, causing a more significant phytotoxic impact on seedlings from desiccated plants. In addition to absorption, the translocation of herbicides can also mediate lethal effects, as this translocation may be limited or favored by environmental factors at the time of application.^48^ These effects include activating the antioxidant defense of plants. Increased CAT activity may indicate increased stress and the ability to adapt through ROS detoxification.^57^ Sinegovskaya and Dushko,^58^ investigating the role of enzymes in increasing soybean plant resistance to herbicides, state that the increase in CAT activity is related to the oxidative stress provided by herbicides, and this increase is essential for plant resistance.

**Figure 4:**
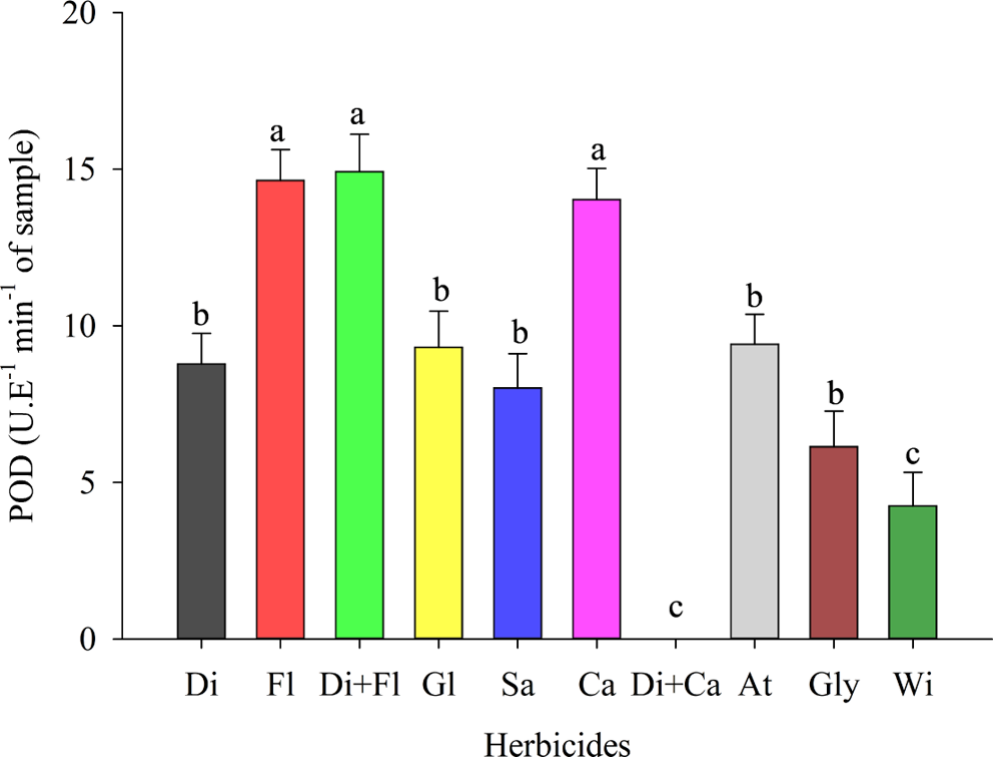
Peroxidase (POD) in cowpea (BRS Tumucumaque) seedlings desiccated with herbicides in preharvest. Di: Diquat; Fl: Flumioxazin; Di + Fl: diquat + flumioxazin; Gl: Glufosinate; Sa: Saflufenacil; Ca: Carfentrazone; Di + Ca: diquat + carfentrazone; At: Atrazine; Gly: Glyphosate; Wi: witness. * Means followed by the same letter do not differ from each other using the Scott-Knott test at 5% probability.

For the peroxidase enzyme (POD), a behavior similar to that of CAT was observed, with the highest values obtained in plants submitted to the application of diquat + flumioxazin (T3) and flumioxazin (T2); the increase observed was 71.56 and 71.01%, respectively (Figure 4).

POD is considered an indicator of biotic and abiotic stress, which detoxifies H_2_O_2_ in the plant.^59^ Given this aspect, herbicide residues can stress seeds and seedlings from desiccated plants.^3^ A high POD activity is correlated with oxidative stress, i.e., with the presence of EROS. Therefore, ROS can be harmful because it causes lipid peroxidation but can also serve as a signaling factor for stress conditions.^60^ Fakhari et al.,^27^ in their study with wheat, state that this stress caused by herbicides depends on parameters such as environmental conditions (time of application), the affected plant tissue, and the mechanism of action of the herbicide. Notably, these factors denote that preharvest cowpea desiccation can directly interfere with the physiological quality of the seed and cause the formation of low-quality seedlings or even generate abnormal seedlings.^3,61^ However, recent research has demonstrated quality data on seedling morphological characteristics, membrane integrity through electrical conductivity, and germination parameters.^34^ Therefore, it is essential to investigate the physiological quality mediated by CAT, POD and PPO activity data.

**Figure 5:**
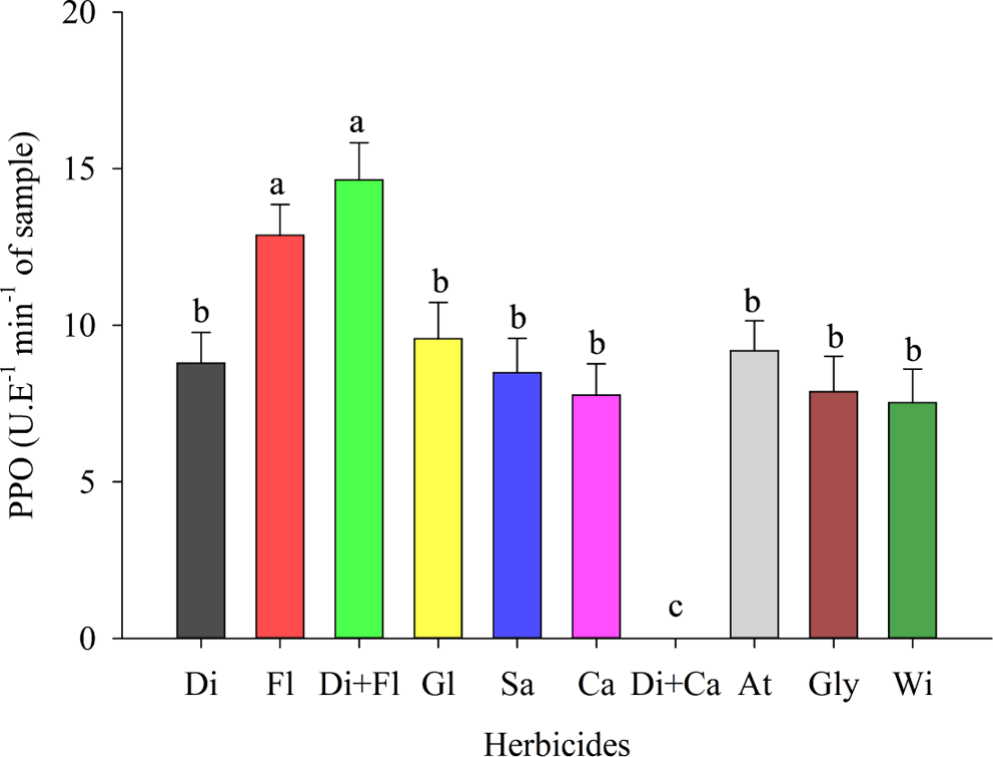
Polyphenoloxidase (PPO) in cowpea (BRS Tumucumaque) seedlings desiccated with herbicides in preharvest. Di: Diquat; Fl: Flumioxazin; Di + Fl: diquat + flumioxazin; Gl: Glufosinate; Sa: Saflufenacil; Ca: Carfentrazone; Di + Ca: diquat + carfentrazone; At: Atrazine; Gly: Glyphosate; Wi: witness. * Means followed by the same letter do not differ from each other using the Scott-Knott test at 5% probability.

Polyphenoloxidase (PPO) activity was also higher in the previously mentioned treatments, specifically in T3 and T2 (diquat + flumioxazin and flumioxazin) (Figure 5). The other treatments did not show significant differences for this enzyme (Figure 5). PPO is an enzyme that catalyzes the oxidation of phenolic compounds in quinones, which promotes the production of pigments that cause darkening in damaged tissues.^62^ Therefore, the higher activity of this enzyme shows that the use of diquat + flumioxazin and flumioxazin for cowpea desiccation can reduce postharvest quality and tissue integrity since the action of PPO reduces nutritional quality and alters flavor.^63^ The reduction of PPO in the control treatment and in plants desiccated with the other herbicides (T10, T4, T5, T6, T7, T8, T9 and T1) can be explained by the suppression of PPO caused by the increase in phenolic compounds, which act as nonenzymatic antioxidant compounds and as inhibitors of PPO.^64^

### 2.2 Experiment II

The interaction between application schedules and herbicides was significant for chlorophyll a, chlorophyll b, total chlorophyll, carotenoids and chlorophyll a/b ratio (Table 2). The control treatment differed significantly from the other treatments for chlorophyll a, chlorophyll b, total chlorophyll and carotenoids (Table 2).

**Table 2:** Analysis of Variance of Chlorophyll *a*, Chlorophyll *b*, Total Chlorophyll, Chlorophyll *a*/*b* Ratio (Chlo *a*/*b*), and Carotenoids from Cowpea Seedlings (BRS Tumucumaque) Desiccated with Herbicides in the Preharvest at Different Times^a^

	F test				
sources of variation	chlorophyll *a* (mg g^–1^)	chlorophyll *b* (mg g^–1^)	total chlorophyll total (mg g^–1^)	chlo *a*/*b* (mg g^–1^)	carotenoids (mg g^–1^)
times (T)	150.31^b^	10.54^b^	128.55^b^	8.97^b^	11.94^b^
desiccants (D)	4.89^c^	4.50^c^	4.87^c^	4.05^c^	61.77^b^
T x D	5.01^b^	3.99^c^	3.63^c^	4.76^b^	42.59^b^
witness × factorial	417.83^b^	218.55^b^	567.24^b^	2.71	56.01^b^
CV (%)	4.28	7.37	3.92	9.26	7.55

a CV: coefficient of variation; ns, not significant.

b Significant at 1% probability by F test.

c Significant at 5% probability by F test.

The application of desiccant herbicides at all times tested caused a reduction in chlorophyll a, so it was possible to verify a reduction of up to 50.23% with the use of the mixture of diquat + flumioxazin at 12 p.m. (Table 3). Among the application times, it can be observed that the lowest damage to chlorophyll a was at 6 a.m. (Table 3). For chlorophyll b, the same trend was observed, and in plants treated with diquat + carfentrazone at 12 p.m., the lowest value was obtained, causing a reduction of 55.19% (Table 3).

**Table 3:** Concentration of Chlorophyll *a*, Chlorophyll *b*, Total Chlorophyll, Ratio between Chlorophyll *a* and *b* (Chlo *a*/*b*), and Carotenoids from Cowpea (BRS Tumucumaque) Seedlings Desiccated with Herbicides in the Preharvest at Different Times^a^

		times (h)		
variables	herbicides	6 a.m.	12 p.m.	6 p.m.
chlorophyll *a* (mg g^–1^)	diquat	12.32 bA	9.55 aC	10.88 aB
	diquat + carfentrazone	13.88 aA	9.37 aC	11.49 aB
	diquat + flumioxazin	14.18 aA	8.84 aC	11.69 aB
	witness		17.76 α	
chlorophyll *b* (mg g^–1^)	diquat	4.54 aA	3.99 bA	3.99 aA
	diquat + carfentrazone	4.58 aA	3.24 cB	4.21 aA
	diquat + flumioxazin	4.76 aA	4.56 aA	4.11 aB
	witness		7.23 α	
total chlorophyll (mg g^–1^)	diquat	16.86 bA	13.55 aC	14.88 aB
	diquat + carfentrazone	18.47 aA	12.61 aC	15.71 aB
	diquat + flumioxazin	18.94 aA	13.41 aC	15.80 aB
	witness		24.98 α	
chlo *a*/*b* (mg g^–1^)	diquat	2.72 aA α	2.41 bA α	2.74 aA α
	diquat + carfentrazone	3.03 aA α	2.93 aA α	2.72 aA α
	diquat + flumioxazin	2.98 aA α	1.94 cB α	2.85 aA α
	witness		2.45 α	
carotenoids (mg g^–1^)	diquat	0.48 bAB	0.41 cB α	0.52 bA
	diquat + carfentrazone	0.48 bC	0.91 aA	0.66 aB
	diquat + flumioxazin	0.60 aA	0.52 bB	0.48 bB
	witness		0.37 α	

a Means followed by equal lowercase letters in the columns and averages followed by equal uppercase letters in the rows do not differ from each other by the Scott-Knott test at 5% probability; Means followed by “α” do not differ from the control by Dunnett’s test at 5% probability.

Diquat is an herbicide of the bipyridyl group that acts by diverting the flow of electrons from photosystem I (PSI) and competes with ferredoxin to bind to PSI.^65^ From this, a reaction of these electrons with molecular oxygen occurs and the formation of reactive oxygen species (ROS) such as superoxide anion (O_2_^•–^), hydroxyl radical (OH^•^) and hydrogen peroxide (H_2_O_2_).^36^ The cell death caused by diquat is due to the accumulation of EROS, thus generating oxidative stress that drives lipid peroxidation.^66^ In contrast, flumioxazin and carfentrazone act by inhibiting the enzyme protoporphyrinogen oxidase (PROTOX), which is involved in chlorophyll biosynthesis by catalyzing the oxidation of protoporphyrinogen IX to protoporphyrin IX.^40^ With the action of PROTOX-inhibiting herbicides, the accumulation of protoporphyrin IX in the cytosol occurs.^67^ In general, all the herbicides used in this experiment alter the photosynthetic mechanism by acting on the transport of electrons in the photochemical phase or on the formation of essential photosynthetic pigments.

Total chlorophyll was also reduced with the application of desiccants so that the lowest values obtained were at 12 and 6 p.m. (Table 3). At these times, it was possible to observe a reduction of 49.36% with the application of diquat + carfentrazone (12 p.m.) and of 40.44% with the application of diquat (6 p.m.) (Table 3). For the chlorophyll a/b ratio, no significant differences could be found (Table 3).

Notably, herbicides applied in full sun and at night have a distinct translocation and efficacy for weed control. For desiccation, the results indicate that the application time can also influence the physiological and biochemical characteristics of desiccated plant seedlings. Diquat and PROTOX inhibitor herbicides directly affect the photosynthetic process and consequently cause chlorophyll reduction. In general, the effects of herbicides are correlated with translocation, which in turn are mediated by factors inherent to plants and environmental factors.^68^

The highest carotenoids values were obtained with the application of diquat + carfentrazone at 12 and 6 p.m. (Table 3). The lowest carotenoid value was observed in the control treatment, and the increase observed in the most severe treatments was 59.35 and 43.94%, respectively (Table 3).

The increase in carotenoids is associated with acclimatization to stress, so control treatment plants usually have lower carotenoid values. The results obtained for carotenoids confirm that the application of herbicides may have caused oxidative stress in the seedlings. In their study, Kolašinac et al.^69^ highlight the increase of carotenoids under stress conditions as a plant response strategy and due to their involvement in the signaling process.

The interaction between application schedules and herbicides was significant for total soluble sugars and proline (Table 4). The control treatment differed significantly from the other treatments for total soluble sugars and proline (Table 4).

**Table 4:** Analysis of Variance of the Concentration of Total Soluble Sugars (TSS) and Proline in Cowpea (BRS Tumucumaque) Seedlings Submitted to Preharvest Herbicide Application at Different Times^a^

	F test	
sources of variation	TSS (mg g^–1^)	Proline (mg g^–1^)
times (T)	128.93^b^	65.77^b^
desiccants (D)	11.49^b^	4.63^c^
T × D	12.26^b^	18.83^b^
witness × factorial	1041.96^b^	40.98^b^
CV (%)	1.61	7.08

a CV: coefficient of variation; ns: not significant.

b Significant at 1% probability by F test.

c Significant at 5% probability by F test.

It was possible to observe that the total soluble sugar (TSS) content was also reduced by the application of herbicides at different times (Table 5). In addition, it was found that the applications at 12 p.m. and 6 p.m. caused more severe damage to the TSS (Table 5). The mixture diquat + flumioxazin provided the lowest TSS value at 6 p.m., with a reduction of 27.74% (Table 5).

**Table 5:** Concentration of Total Soluble Sugars (TSS) and Proline in Cowpea (BRS Tumucumaque) Seedlings Desiccated with Herbicides in the Preharvest at Different Times^a^

		times (h)		
variables	herbicides	6 a.m.	12 p.m.	6 p.m.
TSS (mg g^–1^)	diquat	0.006345 cA	0.006064 aB	0.006036 aB
	diquat + carfentrazone	0.006794 bA	0.006070 aB	0.006032 aB
	diquat + flumioxazin	0.007064 aA	0.006055 aB	0.006027 aB
	witness		0.00834 α	
proline (mg g^–1^)	diquat	0.0638 aB α	0.0804 bA	0.0824 aA
	diquat + carfentrazone	0.0654 aB α	0.1092 aA	0.0637 bB α
	diquat + flumioxazin	0.0695 aB	0.0859 bA	0.0604 bC α
	witness		0.0553 α	

a Means followed by equal lowercase letters in the columns and averages followed by equal uppercase letters in the rows do not differ from each other by the Scott-Knott test at 5% probability; Means followed by α do not differ from the control by Dunnett’s test at 5% probability.

In the present study, the reduction of sugars confirms the destabilization of the photosynthetic process caused by the herbicides since the chemical desiccation at 12 and 6 p.m. with diquat and its mixtures (carfentrazone and flumioxazin) also reduced chlorophylls. The mechanism of action of the herbicides tested can explain this fact. Thus, the observed reduction in the biosynthesis of these sugars^70^ can be explained through the inhibition of photosystem I and the consequent reduction of photosynthesis caused by diquat, as well as by the inhibition of protoporphyrinogen oxidase, an enzyme essential to the biosynthesis of chlorophyll that is inhibited by the application of PROTOS-inhibiting herbicides.^71^

Proline was lower in the control treatment (Table 5). The highest values of proline were in the herbicides applied at 12 p.m., and the isolated application of diquat at 6 p.m. also caused an increase in the proline content (Table 5). Diquat + carfentrazone applied at 12 p.m. provided an increase of 49.36%, being the highest value recorded (Table 5).

Proline tends to be higher in stressed plants, as do carotenoids. Proline acts in signaling and modulating responses involved in cellular functions and gene expression associated with plants’ adaptation capacity.^72^ In the present study, the accumulation of proline observed at the times that caused the most damage to the seedlings strengthens the assumption that under stress conditions, the accumulation of this osmolyte can occur.^73^

## 3 Conclusions

The contact herbicides used in this study are characterized by the light requirement for their total activity, quickly evidencing the symptoms at the contact points and limiting their damage to their target. However, there are indications that the absorption and translocation of herbicides are favored by nocturnal application, generating a more significant movement of the herbicide in the plant and increasing the damage to the other tissues. For better results and explanations, studies are needed to apply the radiolabeled herbicide, condition the plants to the dark, and observe how the translocation of these herbicides occurs. The test at 12 p.m. confirmed the unfeasibility of herbicide applications during this period. Notably, desiccant herbicides caused oxidative stress, which was affirmed by the increase in CAT, POD, and PPO activity and the increase of carotenoids and proline. The data obtained in the present study serve as indicators for the choice of herbicides that can be used for desiccation to use the seeds for successive cultivation in cowpea crops.

## 4 Methods

### 4.1 Location and Characterization of the Area

The experiments were conducted in the field located in the Didactic Garden, belonging to the Federal Rural University of the Semi-Arid (UFERSA), Mossoró-RN. Experiment I (Exp. I) was conducted from June to August 2022, and Experiment II (Exp. II) was conducted between October and December 2022. According to Köppen, the region’s climate is classified as BSh,^74^ with average annual temperatures of 27.8 °C and annual rainfall of approximately 555 mm. During the period of conduction of the experiments, the accumulated rainfall was 6.35 mm (Exp. I) and 78.4 mm (Exp. II). The average temperature was 28.5 °C (Exp. 1) and 29.6 °C (Exp. II), obtaining the data collected at the Automatic Meteorological Station of the Engineering Center (UFERSA).

The area’s soil is classified as Eutrophic Red-Yellow Ultisol.^75^ To chemically characterize the soil before the experiments were implemented, 15 simple samples were collected at depths of 0.2 and 0.4 m. Then, the samples were homogenized to obtain a composite sample, the results of which are presented in Table 6.

**Table 6:** Chemical Characterization of the Soil at Depths of 0.2 and 0.4 m in the Experimental Area

	pH	CE	P	K^+^	Na^+^	Ca^2+^	Mg^2+^
	H_2_O	dS m^–1^	mg dm^–3^			cmol_c_ dm^–3^	
0.2 m	7.56	0.08	156.77	156.00	15.20	3.50	0.86
0.4 m	7.45	0.05	106.23	145.87	15.20	3.00	0.43

The useful area of the plot was composed of four rows of 4.0 m in length, with a spacing of 0.5 m between them and 0.2 m between plants. Four seeds per hole were used for sowing, and thinning was done 14 days after planting, leaving only two plants per hole. The cultivar used was BRS Tumucumaque, characterized by having a semierect size, a cycle of 70 to 75 days and good productive potential.^76^

According to the technical recommendations, we carry out the cultural treatments during the crop development cycle. Topdressing was done at 30 days after sowing (DAS) with the application of 30 kg of N ha^–1^, 15 kg of P ha^–1^ and 10 kg of K ha^–1^, using urea (45% of N), monoammonium phosphate (54% of P_2_O_5_) and potassium chloride (60% of K_2_O).

We perform manual weeding for weed management according to the degree of infestation. Phytosanitary management was carried out with two applications of the insecticide Connect at a dose of 700 mL ha^–1^.

### 4.2 Experiment I

#### 4.2.1 Treatments and Experimental Design

The experiment was arranged in a randomized block design, with three replications and ten treatments. The following treatments were tested: (1) diquat, (2) flumioxazin, (3) diquat + flumioxazin, (4) glufosinate ammonium, (5) saflufenacil, (6) carfentrazone, (7) diquat + carfentrazone, (8) atrazine, (9) glyphosate, (10) control without application. The characterization of the herbicides used in the experiment is presented in Table 7.

**Table 7:** Characterization of Herbicides and Doses Used in the Experiment

active ingredient	commercial product	commercial product dose	applied dose of active ingredient (a.i.)/acid equivalent (a.e.)
diquat	reglone	2 L ha^–1^	400 g i.a ha^–1^
flumioxazin	sumyzin	0.5 L ha^–1^	25 g i.a ha^–1^^a^
diquat + flumioxazin	reglone + sumyzin500	2 L ha^–1^ + 0.5 L ha^–1^	400 g i.a ha^–1^ + 25 g i.a ha^–1^^a^
glufosinate	fascinate BR	2 L ha^–1^	400 g i.a ha^–1^
saflufenacil	heat	140 g ha^–1^	98 g i.a ha^–1^
carfentrazone-ethyl	aurora	0.125 L ha^–1^	50 g i.a ha^–1^^a^
diquat	reglone	2 L ha^–1^	400 g i.a ha^–1^^a^
carfentrazone	aurora	0.125 L ha^–1^	50 g i.a ha^–1^
atrazine	herbitrin	5 L ha^–1^	2.5 g i.a ha^–1^^a^
glyphosate	roundup original DI	4 L ha^–1^	1.480 g e.a ha^–1^

a Applied dose of active ingredient (a.i.)/acid equivalent (a.e.).

#### 2.2.2 Herbicide Application

The herbicides were applied to the 65 DAS using a CO_2_ pressurized knapsack sprayer with two Teejet TT11002 spray tips, air induction and a pressure of 3 bar. The spray volume was 200 L ha^–1^, and the applications were conducted between 7:00 and 8:00 p.m. The choice of application time was based on the frequency with which nighttime application is used in seed production fields. The climatic conditions at the time of application were wind speed of 3.24 m/s and relative humidity of 71.17% (Automatic Weather Station).

The time of days of anticipation of harvest was determined by the number of days between herbicide application and harvest, based on the adequate time for harvest of the control treatment (control).

### 4.3 Experiment II

#### 4.3.1 Treatments and Experimental Design

The experimental design was in randomized blocks in a 3 × 3 + 1 factorial scheme with three replications. The treatments were combinations of three herbicides (diquat, diquat + carfentrazone and diquat + flumioxazin) and three application times (6 a.m., 12 p.m. and 6 p.m.), with the additional treatment consisting of a control without application.

#### 4.3.2 Herbicide Application

The herbicides were applied to the 71 DAS using a CO_2_ pressurized pressure knapsack sprayer with two spray tips of the TT11002 model with air induction and a pressure of 2.5 bar. The spray volume was 200 L ha^–1^. During the application times, the weather conditions were as follows: wind speed of 1.78 m/s (6 a.m.), 3.51 m/s (12 p.m.) and 3.70 m/s (6 p.m.), respectively, and relative humidity of 84.2% (6 a.m.), 40.0% (12 p.m.) and 65.58% (6 p.m.), respectively.

### 4.4 Preparation of the Plant Extract

A total of 10 samples of normal seedlings present in each replication were collected and packed in plastic bags and stored in a freezer (−10 °C). The preparation of the plant extract required for the biochemical and enzymatic tests was done by weighing 0.2 g of fresh seedling mass and placing them in hermetically sealed tubes with the addition of 3 mL of 60% alcohol. Then, the maceration of the plant material was carried out, and the tubes were placed in a water bath at 60 °C for 20 min and then subjected to centrifugation. After the centrifugation process, the supernatant was collected to measure biochemical variables.

### 4.5 Variables Analyzed

#### 4.5.1 Experiment I

##### 4.5.1.1 Total Soluble Amino Acids

For the quantification of total amino acids (TSAA), the acid ninhydrin method.^77^ Glycine was used as the standard substance of the curve. The solution test tubes were stirred and taken to the water bath at 100 °C for 20 min. Then, 60% ethanol was added, and the tubes were stirred again. The readings were performed in a spectrophotometer at 570 nm, and the results were expressed in μmol TSAA g^–1^ of fresh mass.

##### 4.5.1.2 Determination and Extraction of Protein

For protein extraction, frozen tissue samples (0.5 g) added with 25 mg poly(vinylpyrrolidone) (PVP) were macerated in liquid nitrogen and extracted with 0.1 mM acetate buffer (pH 5.0), containing 0.5 mL of 0.1 mM ethylenediaminetetraacetic acid (EDTA). The extracts were centrifuged at 10,000 rpm at 4 °C for 10 min, and the supernatant was used to determine soluble proteins according to Bradford^78^ and enzymatic activities. The readings were made in a spectrophotometer at 595 nm to quantify proteins. The results were expressed in mg g^–1^.

##### 4.5.1.3 Catalase

According to Havir and McHale,^79^ catalase activity was determined by spectrophotometry with modifications by Azevedo et al.^80^ The catalase assay was performed in a solution containing 2.75 mL of 100 mM potassium phosphate buffer (pH 7.5), 100 μL of protein extract, and 120 μL of H_2_O_2_ solution. Next, the H_2_O_2_ consumption was determined based on the decrease in absorbance at 240 nm for 1 min. The results were expressed in μmol/min/mg prot.

##### 4.5.1.4 Peroxidase

Twenty-five μL of guaiacol (0.2 M), 250 μL of hydrogen peroxide (0.38 M) and 1 mL of sodium phosphate buffer (0.2 M pH 6.0) were added to Eppendorf tubes. The tubes were shaken, and the enzymatic reaction was initiated by adding 25 μL of the protein extract. The readings were taken in a spectrophotometer at 470 nm and interspersed for 10 s for 1 min. The results were expressed in E.U.^–1^ min^–1^ of the sample.^81^

##### 4.5.1.5 Polyphenoloxidase

Polyphenoloxidase activity was determined according to the methodology proposed by Campos et al.^82^ A solution containing 1.8 mL of potassium phosphate buffer (0.05 M pH 6.0), 50 μL of protein extract, and 50 μL of catechol (0.1 M) were added to cryogenic tubes. The tubes were vortexed and incubated for 30 min at 30 °C, then 100 μL of perchloric acid was added. The readings were performed in a spectrophotometer at 395 nm, and the results were expressed in E.U.^1–^ min^–1^ of the sample.^81^

#### 4.5.2 Experiment II

##### 4.5.2.1 Chlorophyll and Carotenoids

The chlorophyll content was measured by weighing 0.2 g of fresh matter placed in hermetically sealed test tubes and adding 10 mL of 80% acetone. The tubes were kept for 24 h in an ultrafreezer. After this period, the extracts were placed in cuvettes, and a spectrophotometer was read with absorbances at 645, 652, and 663 nm for chlorophylls and 470 nm for carotenoids.^83^ With the readings, chlorophylls a, b, a + b and total,^84^ carotenoids^85^ and the ratio of chlorophyll a/b were measured. The results were expressed in mg per gram of fresh weight of leaf tissue (mg g^–1^).

##### 4.5.2.2 Total Soluble Sugars

The soluble sugar content (TSS) was determined by the anthrone method.^86^ An aliquot of 50 μL of the plant extract was used for this analysis, plus 950 mL of distilled water. Then, the tubes were placed in an ice bath while 2 mL of anthrone was added. The tubes were swirled in a vortex and placed back in the ice bath and then in the water bath for 8 min. The readings were taken in a spectrophotometer at 620 nm. The results were expressed in mg of TSS g^–1^ of fresh mass.

##### 4.5.2.3 Proline

The quantification of proline was performed using the methodology proposed by Bates et al.,^87^ placing 1 mL of plant extract, 1 mL of acid ninhydrin and 1 mL of glacial acetic acid in test tubes and shaking them. Then, the tubes were placed in a water bath at 100 °C for 1 h. After this period, the tubes were cooled in an ice bath, and 2 mL of toluene was added to shake them again. Aspiration was performed with a Pasteur pipet, and the reading was performed in a spectrophotometer at 520 nm. The results were expressed in mg g^–1^ of fresh matter.

### 4.6 Data Analysis

The normality of the data was verified by the Shapiro-Wilk test, and the Barlett test was used to verify the homoscedasticity of the residuals. In Experiment I, the data were submitted for analysis of variance (F test), and the means were compared using the Scott-Knott test (5% probability). In Experiment II, the data were submitted to analysis of variance (F test) and the means were compared with each other by the Scott-Knott test to compare the means within each factor or to unfold the significant interactions, and the Dunnett test was used to compare the control and the other treatments, both at 5% probability. The analyses were performed using the R software.^88^

## References

[ref1] Raisse E. R.; Assis M. D. O.; Araujo E. F.; Freitas F. C. L. D. E.; Araujo R. F. Chemical desiccants for anticipation of harvest and physiological quality of cowpea seeds. Rev. Caatinga 2020, 33, 878–887. 10.1590/1983-21252020v33n402rc.

[ref2] Follmann D. N.; Cargnelutti Filho A.; de Souza V. Q.; Nardino M.; Carvalho I. R.; Demari G. H.; Ferrari M.; de Pelegrin A. J.; Szareski V. J. Relações lineares entre caracteres de soja safrinha. Rev. Ciênc. Agr. 2017, 40 (1), 213–221. 10.19084/RCA16027.

[ref3] Monçon Fipke G.; Fipke G. M.; Martin T. N.; Nunes U. R.; Deak v. A.; Stecca J. D. L.; Leivas Stecca J. D.; Winck J. E. M.; Minussi Winck J. E.; Grando L. F. T.; Teleken Grando L. F.; da Costa Rossato A. Application of non-selective herbicides in the pre-harvest of wheat damages seed quality. Am. J. Plant Sci. 2018, 09 (1), 107–123. 10.4236/ajps.2018.91010.

[ref4] Peske S. T.; Villela F. A.; Meneghello G. E. Sementes: fundamentos científicos e tecnológicos; Editora Universitária/UFPel: Pelotas, 2012.

[ref5] Subedi M.; Willenborg C. J.; Vandenberg A. Influence of harvest aid herbicides on seed germination, seedling vigor and milling quality traits of red lentil (Lens culinaris L.). Front. Plant Sci. 2017, 8, 247150 10.3389/fpls.2017.00311. PMC5349153 28352275

[ref6] Zanatta T. P.; Kulczynski S. M.; Della Libera D.; Testa V.; Fontana D. C.; Werner C. J.; Ballen E. M. Produtividade e qualidade fisiológica de sementes de soja colhidas em diferentes períodos de maturação. Rev. Cultivando o Saber 2018, 11 (1), 89–106.

[ref7] Assis M. O.; Araujo E. F.; Freitas F. C. L.; Silva L. J.; Araujo R. F. Pre-harvest desiccation in productivity and physiological quality of cowpea seeds. Planta Daninha 2019, 37, e019177741 10.1590/s0100-83582019370100014.

[ref8] Andrade Junior A. S.; Bastos E. A.; Monteiro J. E. Zoneamento agrícola de risco climático para o feijão-caupi em cultivo convencional e plantio direto no estado do Piauí, 2018.

[ref9] Wijewardana C.; Reddy K. R.; Krutz L. J.; Gao W.; Bellaloui N. Drought stress has transgenerational effects on soybean seed germination and seedling vigor. PLoS One 2019, 14 (9), e0214977 10.1371/journal.pone.0214977. 31498795 PMC6733489

[ref10] Vargas R. L. d.; Schuch L. O.; Barros W. S.; Rigo G. A.; Szareski V. J.; Carvalho I. R.; Pimentel J. R.; Troyjack C.; Jaques L. B.; De Souza V. Q.; et al. Macronutrients and micronutrients variability in soybean seeds. J. Agric. Sci. 2018, 10, 209–222. 10.5539/jas.v10n4p209.

[ref11] Huang Y.; Zhan H.; Bhatt P.; Chen S. Paraquat degradation from contaminated environments: current achievements and perspectives. Front. Microbiol. 2019, 10, 460070 10.3389/fmicb.2019.01754. PMC6689968 31428067

[ref12] Silva P. V. d.; Mendonça Bezera M.; Medeiros E. S. d.; Carvalho Dambrós T.; Mauad M.; Monquero P. A.; Alves Nunes F.; Schedenffeldt B. F. Pre-harvest desiccation strategies of soybean culture: a scenario without paraquat. J. Environ. Sci. Health, Part B 2022, 57 (9), 710–719. 10.1080/03601234.2022.2100680. 35861133

[ref13] ANVISA. Consulta Pública n° 72, de 29 de setembro de 2017 Agência Nacional de Vigilância Sanitária, Diário Oficial da União: Brasília, DF, 2017.

[ref14] McNaughton K. E.; Blackshaw R. E.; Waddell K. A.; Gulden R. H.; Sikkema P. H.; Gillard C. L. Effect of application timing of glyphosate and saflufenacil as desiccants in dry edible bean (Phaseolus vulgaris L.). Can. J. Plant Sci. 2015, 95 (2), 369–375. 10.4141/cjps-2014-157.

[ref15] Jaskulski D.; Jaskulska I. The effect of pre-harvest glyphosate application on grain quality and volunteer winter wheat. Rom. Agric. Res. 2014, 283–289.

[ref16] Zuffo A. M.; Aguilera J. G.; Carvalho E. R.; Teodoro P. E. Harvest times with chemical desiccation and the effects on the enzymatic expression and physiological quality of soybean seeds. Rev. Caatinga 2020, 33, 361–370. 10.1590/1983-21252020v33n209rc.

[ref17] Agrofit. Agrofit. Ministério da Agricultura; Pecuária e Abastecimento, 2021.

[ref18] Finoto E. L.; Sediyama T.; Alves de Albuquerque J. d. A.; Bernades Soares M. B.; Altafin Galli J.; Cordeiro Junior P. S.; Santos de Menezes P. H. Antecipação e retardamento de colheita nos teores de óleo e proteína das sementes de soja, cultivar Valiosa RR. Sci. Agropecu. 2017, 8 (2), 99–107.

[ref19] Maciel C. D. d. G.; Iuchemin C. E. L.; de Souza M. V.; da Silva A. A. P.; Karpinski R. A. K.; Helvig E. O.; Karpinski P. K. K.; Baixo B. T.; Matias J. P. Control efficiency of black bindweed by glyphosate and glyphosate+ 2, 4-D on different application times. Rev. Bras. Herbic. 2016, 15 (4), 380–387.

[ref20] Inomjonovich S. R.; Akbaralievich M. A.; Karimov N. D. Preliminary results of the use of herbicide in the fight against weeds (grainy) in the field of soybeans. J. Adv. Zool. 2023, 44, 110–114.

[ref21] Roman E. S.; Beckie H.; Vargas L.; Hall L.; Rizzardi M. A.; Wolf T. M. Como funcionam os herbicidas: da biologia à aplicação; Berthier Passo Fundo, 2007.

[ref22] Cieslik L. F.; Vidal R. A.; Trezzi M. M. Fatores ambientais que afetam a eficácia de herbicidas inibidores da ACCase: Revisão. Planta Daninha 2013, 31, 483–489. 10.1590/S0100-83582013000200026.

[ref23] Correia N. M. Comportamento dos herbicidas no ambiente; EMBRAPA, 2018.

[ref24] Brochado M. G. d. S.; Guidi Y. M.; Lima A. d. C.; Medeiros B. A. d. P.; D’Angieri R.; Mendes K. F. Can herbicides of different mode of action cause injury symptoms in non-herbicide-tolerant young soybean due to simulated drift?. J. Environ. Sci. Health, Part B 2023, 58 (12), 726–743. 10.1080/03601234.2023.2275512. 37904543

[ref25] Rubenich R.; Schaedler C. E.; Zandoná R. R.; de Melo Scalcon R.; Chiapinotto D. M. Efeito da reduÃ § Ã£ o de luz na seletividade a herbicidas e rendimento de grÃ£ os do trigo. Rev. Bras. Herbic. 2017, 16 (4), 296–306.

[ref26] Schneider M. V.; Rosa M. F.; Lobo V. d. S.; Bariccatti R. A. Degradação fotocalítica de bentazona com TiO 2. Eng. Sanit. Ambiental 2014, 19, 61–66. 10.1590/S1413-41522014000100007.

[ref27] Fakhari R.; Tobeh A.; Alebrahim M. T.; Mehdizadeh M.; Khiavi H. K. Study of changes in activity of wheat antioxidant enzymes under stress residue of imazethapyr herbicide. Int. J. Adv. Biol. Biomed. Res. 2020, 8 (2), 165–179. 10.33945/SAMI/IJABBR.2020.2.7.

[ref28] Hanafy R. S.; Sadak M. S. Foliar spray of stigmasterol regulates physiological processes and antioxidant mechanisms to improve yield and quality of sunflower under drought stress. J. Soil Sci. Plant Nutr. 2023, 23 (2), 2433–2450. 10.1007/s42729-023-01197-4.

[ref29] Sadak M. S.; Hanafy R. S.; Elkady F. M. A. M.; Mogazy A. M.; Abdelhamid M. T. Exogenous calcium reinforces photosynthetic pigment content and osmolyte, enzymatic, and non-enzymatic antioxidants abundance and alleviates salt stress in bread wheat. Plants 2023, 12 (7), 1532 10.3390/plants12071532. 37050158 PMC10097001

[ref30] El-Bassiouny H.; Sadak M. S.; Mahfouz S.; El-Enany M.; Elewa T. Use of thiamine, pyridoxine and bio stimulant for better yield of wheat plants under water stress: growth, osmoregulations, antioxidantive defence and protein pattern. Egypt. J. Chem. 2023, 66 (4), 407–424. 10.21608/ejchem.2022.160140.6898.

[ref31] Ould said C.; Boulahia K.; Eid M. A. M.; Rady M. M.; Djebbar R.; Abrous-Belbachir O. Exogenously used proline offers potent antioxidative and osmoprotective strategies to re-balance growth and physio-biochemical attributes in herbicide-stressed Trigonella foenum-graecum. J. Soil Sci. Plant Nutr. 2021, 21, 3254–3268. 10.1007/s42729-021-00604-y.

[ref32] Sadak M. S.; Dawood M. G. Biofertilizer role in alleviating the deleterious effects of salinity on wheat growth and productivity. Gesunde Pflanz. 2023, 75 (4), 1207–1219. 10.1007/s10343-022-00783-3.

[ref33] Silva J. N.; Costa E. M.; Pereira L. S.; Gonçalves E. C. Z.; Zuchi J.; Jakelaitis A. Cowpea yield and quality after application of desiccating herbicides. J. Seed Sci. 2020, 42, e202042019 10.1590/2317-1545v42228204.

[ref34] Chamma L.; Silva G. F. d.; Perissato S. M.; Alievi C.; Chaves P. P. N.; Giandoni V. C. R.; Calonego J. C.; Silva E. A. A. d. Does Forced Plant Maturation by Applying Herbicide with Desiccant Action Influence Seed Longevity in Soybean?. Plants 2023, 12 (15), 2769 10.3390/plants12152769. 37570923 PMC10420660

[ref35] Almeida I. L. d.; Vieira W. F.; Souza N. O. S.; Suinaga F. A.; Amabile R. F.; Fagioli M. Chemical desiccants for anticipation of harvest and quality improvement of chickpea seeds. Hortic. Bras. 2023, 41, e2506 10.1590/s0102-0536-2023-e2506.

[ref36] De Oliveira G. M. P.; Mendes G.; De Aguiar E Silva M. A.; Dalazen G. Growth inhibition of sourgrass as a function of period of darkness after diquat application. Biosci. J. 2022, 38, e38087 10.14393/BJ-v38n0a2022-62470.

[ref37] Pitelli R. A.; Bisigatto A. T.; Kawaguchi I.; Pitelli R. Doses e horário de aplicação do diquat no controle de Eichhornia crassipes. Planta Daninha 2011, 29, 269–277. 10.1590/S0100-83582011000200004.

[ref38] Santos Junior A.; Freitas F. C. L.; Santos I. T.; Silva D. C.; Paixão G. P.; Sediyama C. S. Management of Commelina benghalensis with saflufenacil in shaded environments. Planta Daninha 2019, 37, e019178088 10.1590/s0100-83582019370100051.

[ref39] Soltani N.; Blackshaw R. E.; Gulden R. H.; Gillard C. L.; Shropshire C.; Sikkema P. H. Desiccation in dry edible beans with various herbicides. Can. J. Plant Sci. 2013, 93 (5), 871–877. 10.4141/cjps2013-061.

[ref40] Singh S.; Tiwari S. Responses of Plants to Herbicides: Recent Advances and Future Prospectives. In Plant Life Under Changing Environment; Elsevier, 2020; pp 237–250.

[ref41] Vidal R. A.; Merotto A. Jr; Schaedler C. E.; Pinto Lamego F.; Portugal J.; Menendes J.; Kozlowski L. A.; Muzell Trezzi M.; De Prado R. Mecanismos de ação dos herbicidas. Aspectos da biologia e manejo das plantas daninhas 2014, 10, 235–256.

[ref42] Batista-Silva W.; Heinemann B.; Rugen N.; Nunes-Nesi A.; Araújo W. L.; Braun H. P.; Hildebrandt T. M. The role of amino acid metabolism during abiotic stress release. Plant, Cell Environ. 2019, 42 (5), 1630–1644. 10.1111/pce.13518. 30632176

[ref43] Araújo W. L.; Tohge T.; Ishizaki K.; Leaver C. J.; Fernie A. R. Protein degradation–an alternative respiratory substrate for stressed plants. Trends Plant Sci. 2011, 16 (9), 489–498. 10.1016/j.tplants.2011.05.008. 21684795

[ref44] Signorelli S. The fermentation analogy: a point of view for understanding the intriguing role of proline accumulation in stressed plants. Front. Plant Sci. 2016, 7, 213126 10.3389/fpls.2016.01339. PMC5015475 27642286

[ref45] Hildebrandt T. M. Synthesis versus degradation: directions of amino acid metabolism during Arabidopsis abiotic stress response. Plant Mol. Biol. 2018, 98, 121–135. 10.1007/s11103-018-0767-0. 30143990

[ref46] Chen Y.; Hoehenwarter W. Changes in the phosphoproteome and metabolome link early signaling events to rearrangement of photosynthesis and central metabolism in salinity and oxidative stress response in Arabidopsis. Plant Physiol. 2015, 169 (4), 3021–3033. 10.1104/pp.15.01486. 26471895 PMC4677922

[ref47] Savchenko T.; Tikhonov K. Oxidative stress-induced alteration of plant central metabolism. Life 2021, 11 (4), 304 10.3390/life11040304. 33915958 PMC8066879

[ref48] Hatamleh A. A.; Danish M.; Al-Dosary M. A.; El-Zaidy M.; Ali S. Physiological and oxidative stress responses of Solanum lycopersicum (L.)(tomato) when exposed to different chemical pesticides. RSC Adv. 2022, 12 (12), 7237–7252. 10.1039/D1RA09440H. 35424659 PMC8982233

[ref49] Yilmaz V.; Ari E. The Effects of Service Quality, Image, and Customer Satisfaction on Customer Complaints and Loyalty in High-Speed Rail Service in Turkey: A Proposal of the Structural Equation Model. In Transportmetrica A: Transport Science; Taylor & Francis, 2017; Vol. 13; pp 67–90.

[ref50] Sachdev S.; Ansari S. A.; Ansari M. I.; Fujita M.; Hasanuzzaman M. Abiotic stress and reactive oxygen species: Generation, signaling, and defense mechanisms. Antioxidants 2021, 10 (2), 277 10.3390/antiox10020277. 33670123 PMC7916865

[ref51] Ahmad R.; Hussain S.; Anjum M. A.; Khalid M. F.; Saqib M.; Zakir I.; Hassan A.; Fahad S.; Ahmad S. Oxidative stress and antioxidant defense mechanisms in plants under salt stress. Plant Abiotic Stress Tolerance 2019, 191–205. 10.1007/978-3-030-06118-0_8.

[ref52] Acar A. In vivo toxicological assessment of diquat dibromide: cytotoxic, genotoxic, and biochemical approach. Environ. Sci. Pollut. Res. 2021, 28 (34), 47550–47561. 10.1007/s11356-021-13936-0. 33893917

[ref53] Jiang L.; Yang H. Prometryne-induced oxidative stress and impact on antioxidant enzymes in wheat. Ecotoxicol. Environ. Saf. 2009, 72 (6), 1687–1693. 10.1016/j.ecoenv.2009.04.025. 19473703

[ref54] Zhang J. J.; Lu Y. C.; Zhang J. J.; Tan L. R.; Yang H. Accumulation and toxicological response of atrazine in rice crops. Ecotoxicol. Environ. Saf. 2014, 102, 105–112. 10.1016/j.ecoenv.2013.12.034. 24530725

[ref55] Boulahia K.; Carol P.; Planchais S.; Abrous-Belbachir O. Phaseolus vulgaris L. seedlings exposed to prometryn herbicide contaminated soil trigger an oxidative stress response. J. Agric. Food Chem. 2016, 64 (16), 3150–3160. 10.1021/acs.jafc.6b00328. 27019272

[ref56] Pan D.; Li Q. X.; Lin Z.; Chen Z.; Tang W.; Pan C.; Tan H.; Zeng D. Interactions between salicylic acid and antioxidant enzymes tilting the balance of H2O2 from photorespiration in non-target crops under halosulfuron-methyl stress. Pestic. Biochem. Physiol. 2017, 143, 214–223. 10.1016/j.pestbp.2017.09.007. 29183595

[ref57] Sin’kevich M. S.; Selivanov A. A.; Antipina O. V.; Kropocheva E. V.; Alieva G. P.; Suvorova T. A.; Asta-hova N. V.; Moshkov I. E. Aktivnost’antioksidantnykh fermentov u rastenij Arabidopsis thaliana pri zakalivanii k gipotermii [Activity of antioxidant enzymes of Arabi-dopsis thaliana plants during cold hardening to hypothermia]. Russ. J. Plant Physiol. 2016, 63 (6), 777–782.

[ref58] Sinegovskaya V.; Dushko O. Role of Enzyme Activity in Increasing Soybean Plants’ Resistance to Herbicides; EDP Sciences, 2021; Vol. 254, p 02007.

[ref59] Barbosa M. R.; Silva M. M. d. A.; Willadino L.; Ulisses C.; Camara T. R. Geração e desintoxicação enzimática de espécies reativas de oxigênio em plantas. Cienc. Rural 2014, 44, 453–460. 10.1590/S0103-84782014000300011.

[ref60] Ding G.; Jin Z.; Han Y.; Sun P.; Li G.; Li W. Mitigation of chromium toxicity in Arabidopsis thaliana by sulfur supplementation. Ecotoxicol. Environ. Saf. 2019, 182, 109379 10.1016/j.ecoenv.2019.109379. 31254852

[ref61] Fipke G. M.; Deak E. A.; Stecca J. D. L.; Bernardy D.; Berger M.; Tai L. A.; Martin T. N. Morphology and enzymatic activity of seedlings from wheat desiccated in pre-harvest. Acta Sci., Agron. 2020, 43, e44974 10.4025/actasciagron.v43i1.44974.

[ref62] Queiroz C.; Mendes Lopes M. L.; Fialho E.; Valente-Mesquita V. L. Polyphenol oxidase: characteristics and mechanisms of browning control. Food Rev. Int. 2008, 24 (4), 361–375. 10.1080/87559120802089332.

[ref63] Alvarenga T. C.; da Silva Neto H. F.; Ogassavara F. O.; Arantes F. C.; Marques M. O.; Frigieri M. C. Polifenoloxidase: uma enzima intrigante. Cienc. Tecnol. 2011, 3 ( (1), ).

[ref64] Homayoonzadeh M.; Hosseininaveh V.; Haghighi S. R.; Talebi K.; Roessner U.; Maali-Amiri R. Evaluation of physiological and biochemical responses of pistachio plants (Pistacia vera L.) exposed to pesticides. Ecotoxicology 2021, 30 (6), 1084–1097. 10.1007/s10646-021-02434-1. 34101048

[ref65] Höfer M.; Schäfer M.; Wang Y.; Wink S.; Xu S. Genetic Mechanism of Non-Targeted-Site Resistance to Diquat in Spirodela polyrhiza. Plants 2024, 13 (6), 845 10.3390/plants13060845. 38592881 PMC10975167

[ref66] Chang Z.; Liu Y.; Dong H.; Teng K.; Han L.; Zhang X. Effects of cytokinin and nitrogen on drought tolerance of creeping bentgrass. PLoS One 2016, 11 (4), e0154005 10.1371/journal.pone.0154005. 27099963 PMC4839601

[ref67] Fontes J. R. A.; de Oliveira I. J.; de Morais R. R. Seletividade e eficácia de herbicidas para o controle de plantas daninhas na mandioca, 2021.

[ref68] Takano H. K.; Beffa R.; Preston C.; Westra P.; Dayan F. E. Physiological factors affecting uptake and translocation of glufosinate. J. Agric. Food Chem. 2020, 68 (10), 3026–3032. 10.1021/acs.jafc.9b07046. 32049526

[ref69] Kolašinac S.; Dajić-Stevanović Z.; Kilibarda S. N.; Kostić A. Ž. Carotenoids: New applications of “old” pigments. Phyton 2021, 90 (4), 1041–1062. 10.32604/phyton.2021.015996.

[ref70] Elhakem A. H.; Abd El-Salam M. M. Elimination of the Effect of Some Herbicides on the Growth of Zea mays and Accumulation in the Soil Using Urea. Planta Daninha 2018, 36, e018176075 10.1590/s0100-83582018360100104.

[ref71] Meyers S. L.; Jennings K. M.; Miller D. K.; Shankle M. W. Response of sweetpotato to diquat applied pretransplanting. Weed Technol. 2020, 34 (5), 637–641. 10.1017/wet.2020.27.

[ref72] El Moukhtari A.; Cabassa-Hourton C.; Farissi M.; Savouré A. How does proline treatment promote salt stress tolerance during crop plant development?. Front. Plant Sci. 2020, 11, 553924 10.3389/fpls.2020.01127. PMC7390974 32793273

[ref73] Furlan A. L.; Bianucci E.; Giordano W.; Castro S.; Becker D. F. Proline metabolic dynamics and implications in drought tolerance of peanut plants. Plant Physiol. Biochem. 2020, 151, 566–578. 10.1016/j.plaphy.2020.04.010. 32320942

[ref74] Alvares C. A.; Stape J. L.; Sentelhas P. C.; Gonçalves J. L. d. M.; Sparovek G. Köppen’s climate classification map for Brazil. Meteorol. Z. 2014, 22 (6), 711–728. 10.1127/0941-2948/2013/0507.

[ref75] Embrapa Sistema brasileiro de classificação de solos; Centro Nacional de Pesquisa de Solos: Rio de Janeiro, 2013; Vol. 3.

[ref76] Oliveira I. J.; Fontes J. R. A.; Silva K. J. D.; Rocha M. M. BRS Tumucumaque-cultivar de feijão-caupi com valor nutritivo para o Amazonas, 2014.

[ref77] Yemm E. W.; Cocking E. C.; Ricketts R. E. The determination of amino-acids with ninhydrin. Analyst 1955, 80 (948), 209–214. 10.1039/an9558000209.

[ref78] Bradford M. M. A rapid and sensitive method for the quantitation of microgram quantities of protein utilizing the principle of protein-dye binding. Anal. Biochem. 1976, 72 (1–2), 248–254. 10.1016/0003-2697(76)90527-3. 942051

[ref79] Havir E. A.; McHale N. A. Biochemical and developmental characterization of multiple forms of catalase in tobacco leaves. Plant Physiol. 1987, 84 (2), 450–455. 10.1104/pp.84.2.450. 16665461 PMC1056601

[ref80] Azevedo R. A. d.; Alas R. M.; Smith R. J.; Lea P. J. Response of antioxidant enzymes to transfer from elevated carbon dioxide to air and ozone fumigation, in the leaves and roots of wild-type and a catalase-deficient mutant of barley. Physiol. Plant. 1998, 104 (2), 280–292. 10.1034/j.1399-3054.1998.1040217.x.

[ref81] Bezerra Neto E.; Barreto L. P. Análises químicas e bioquímicas em plantas; UFRPE, Recife, 2011.

[ref82] Campos Â. D.; Ferreira A. G.; Hampe M. M. V.; Antunes I. F.; Brancão N.; Silveira E. P. d.; Osório V. A.; Augustin E. Atividade de peroxidase e polifenoloxidase na resistência do feijão à antracnose. Pesq. Agropec. Bras. 2004, 39, 637–643. 10.1590/S0100-204X2004000700004.

[ref83] Scopel W.; Barbosa J. Z.; Vieira M. L. Extração de pigmentos foliares em plantas de canola; Unoesc & Ciência-ACET, 2011; Vol. 2, pp 87–94.

[ref84] Witham F. H.; Blaydes D. F.; Devlin R. M. Experiments in Plant Physiology, 1971.

[ref85] Lichtenthaler H. K.; Wellburn A. R. Determinations of Total Carotenoids and Chlorophylls a and b of Leaf Extracts in Different Solvents; Portland Press Ltd., 1983.

[ref86] Yemm E. W.; Willis A. J. The estimation of carbohydrates in plant extracts by anthrone. Biochem. J. 1954, 57 (3), 508 10.1042/bj0570508. 13181867 PMC1269789

[ref87] Bates L. S.; Waldren R. P. A.; Teare I. D. Rapid determination of free proline for water-stress studies. Plant Soil 1973, 39, 205–207. 10.1007/BF00018060.

[ref88] Team R. C. R Development Core Team R: A Language and Environment for Statistical Computing 2023; R Foundation for Statistical, 2023.

